# The Attitudes to Ageing Questionnaire: Mokken Scaling Analysis

**DOI:** 10.1371/journal.pone.0099100

**Published:** 2014-06-03

**Authors:** Susan D. Shenkin, Roger Watson, Ken Laidlaw, John M. Starr, Ian J. Deary

**Affiliations:** 1 Department of Geriatric Medicine, University of Edinburgh, Edinburgh, Scotland, United Kingdom; 2 Centre for Cognitive Ageing and Cognitive Epidemiology, Edinburgh, Scotland, United Kingdom; 3 Faculty of Health & Social Care, University of Hull, Hull, England, United Kingdom; 4 Department of Clinical Psychology, University of East Anglia, Norwich, England, United Kingdom; 5 Department of Psychology, University of Edinburgh, Edinburgh, Scotland, United Kingdom; University of Vienna, Austria

## Abstract

**Background:**

Hierarchical scales are useful in understanding the structure of underlying latent traits in many questionnaires. The Attitudes to Ageing Questionnaire (AAQ) explored the attitudes to ageing of older people themselves, and originally described three distinct subscales: (1) Psychosocial Loss (2) Physical Change and (3) Psychological Growth. This study aimed to use Mokken analysis, a method of Item Response Theory, to test for hierarchies within the AAQ and to explore how these relate to underlying latent traits.

**Methods:**

Participants in a longitudinal cohort study, the Lothian Birth Cohort 1936, completed a cross-sectional postal survey. Data from 802 participants were analysed using Mokken Scaling analysis. These results were compared with factor analysis using exploratory structural equation modelling.

**Results:**

Participants were 51.6% male, mean age 74.0 years (SD 0.28). Three scales were identified from 18 of the 24 items: two weak Mokken scales and one moderate Mokken scale. (1) ‘Vitality’ contained a combination of items from all three previously determined factors of the AAQ, with a hierarchy from physical to psychosocial; (2) ‘Legacy’ contained items exclusively from the Psychological Growth scale, with a hierarchy from individual contributions to passing things on; (3) ‘Exclusion’ contained items from the Psychosocial Loss scale, with a hierarchy from general to specific instances. All of the scales were reliable and statistically significant with ‘Legacy’ showing invariant item ordering. The scales correlate as expected with personality, anxiety and depression. Exploratory SEM mostly confirmed the original factor structure.

**Conclusions:**

The concurrent use of factor analysis and Mokken scaling provides additional information about the AAQ. The previously-described factor structure is mostly confirmed. Mokken scaling identifies a new factor relating to vitality, and a hierarchy of responses within three separate scales, referring to vitality, legacy and exclusion. This shows what older people themselves consider important regarding their own ageing.

## Introduction

The population ageing we are witnessing today is unprecedented in the whole of human history [1]. The oldest-old section of society is increasing fastest. For example, in the UK in 1970, there were 1,180 centenarians alive, whereas this figure had increased by almost 12-fold to 12,640 in 2010, projected to increase over twelve-fold to 160,000 by 2035 [2]. A recent report [3] states the UK is “woefully underprepared” for the ageing of society with major changes required to our collective attitudes to ageing. The new cohort of older people may be more different than previous generations and, as such, understanding the attitudes and experiences of ageing of older people is likely to be more rather than less important.

In Western societies, there is a common perception that older people are weak and frail, rather than wise and mature [4]. There is a different more compelling narrative at the individual level, where the trajectory of ageing is experienced as more positive than expected as people report high levels of emotional stability and well-being as the norm [5].

Older people without physical or psychological problems report positive attitudes to ageing [6]. They often do not subscribe to the negative stereotype of ageing, and may reject association with their peer group [4]. Growing older is not defined by chronological age but may be determined by a more personal phemonological experience of ageing [7]. Ageing is more likely a *process* rather than a state, with a great deal of heterogeneity in how people experience ageing. It is, therefore, important that older people's own attitudes to ageing are assessed using well-validated scales incorporating a range of positive and negative attitudes, rather than outdated stereotypical views that many younger people may have about ageing and older people [8]. Previous measures of attitudes to ageing include a five item subscale of the Philadelphia Geriatric Morale Scale [9]. It is widely used and well-constructed but, as a short scale, does not capture all aspects of attitudes to ageing. Some scales have been developed using younger peoples' attitudes to older people, rather than including older people themselves; e.g. Kogan's Attitudes to Older People Questionnaire [10]. Studies often recruit undergraduates as participants [11].

The attitudes to ageing questionnaire (AAQ) [12] was developed to provide a standard way of measuring attitudes to ageing from the perspective of older people. It was part of an international project on Quality of Life of older adults in collaboration with the World Health Organisation (WHO). The 24-item scale incorporates the concepts of both losses and gains with ageing. Factor analysis found three distinct subscales: (1) Psychosocial Loss, (2) Physical Change, and (3) Psychological Growth [12]. Concurrent and discriminant analysis alongside a range of other variables in older people [13] show, for example, that: Psychosocial Loss is positively correlated with neuroticism but negatively correlated with other aspects of personality (extraversion, openness, agreeableness and conscientiousness), positively correlated with anxiety and depression and physical disability; having a positive attitude to Physical Change is negatively correlated with physical disability and social class, negatively correlated with neuroticism and positively correlated with extraversion, openness, agreeableness and conscientiousness, females scored lower on Physical Change than men; Psychological Growth is negatively correlated with depression, negatively correlated with neuroticism and positively correlated with extraversion, openness, agreeableness and conscientiousness [13]. More negative attitudes have been associated with higher scores on scales of depression [14]. The AAQ, and the factor-analytic derived subscale structure, has been validated in Canadian, Norwegian and Spanish samples of older people [14]–[16]. In the Spanish sample the results showed good construct validity, and the AAQ results differed between groups at different levels of education, those with and without depression, comorbidity, and caring responsibility [16]. There were some differences between Canadian and Norwegian respondents [15]: the AAQ was able to distinguish between people who were healthy and those who were not, but the fit statistics of confirmatory factor analysis differed between the groups from the two countries, suggesting that there is a need for further modification and testing of the scale.

Factor analysis, which mainly investigates the relationship between items and total scale scores, has been demonstrated to provide a limited insight into the dimensionality of a scale [17]. Other methods, under the umbrella of item response theory (IRT), offer insight into the properties of individual items, how they function relative to one another and, especially, into whether there is a hierarchy of terms *within* each subscale factor [18]. Demonstrating hierarchies in sets of items from questionnaires is useful because by doing so not only can a score on a questionnaire can be related consistently to a level of the latent trait being measured, but the quantitative level of the trait can be easily related to a qualitative aspect (i.e. the item content). The particular form of IRT used in the present study is non-parametric (Mokken scaling—to be described below) and we have previously used this methodology to determine the hierarchy of items in clinically relevant scales such as the Townsend Functional Ability Scale, the Edinburgh Feeding Evaluation in Dementia scale [19] and the General Health Questionnaire [20]. A relatively non-technical introduction to the method can be found in [19]. This method can, therefore, help us to understand what aspects of attitudes to ageing are most important to older people themselves. It should be noted that a form of IRT (Rasch analysis) was applied in the development of the AAQ [12]. However, it was not used to establish hierarchies in the subscales but to investigate item equivalence across the samples used in the original study and as an additional method of item reduction. Mokken scaling is applied here because it is a different method [21] with specific advantages related to its non-parametric nature, principally, that it is only interested in the ordering of people and items and not on ratio level scores and is, thus, more conservative of items in scales [22].

## Methods

### Ethics Statement

Ethical approval was obtained from the Multi-Centre Ethics Committee for Scotland (MREC/01/0/56) and Lothian Research Ethics Committee (LREC/2003/2/29). The research was conducted in compliance with the Helsinki declaration. All subjects gave written, informed consent.

### The Lothian Birth Cohort 1936

The Scottish Mental Survey of 1947 (SMS1947) applied a valid test of general intelligence to almost all children born in 1936 and attending Scottish schools on 4 June 1947. Between 2004 and 2007, at the Wellcome Trust Clinical Research Facility at the Western General Hospital in Edinburgh, local survivors of the original Scottish Mental Survey of 1947, known as LBC1936 and now aged 70, participated in a re-assessment of the same mental ability test they had originally completed in 1947 at age 11. Between 2004 and 2007, participants completed comprehensive physical and cognitive assessment procedures. In addition, participants took home and returned a large battery of questionnaires assessing personality, anxiety and depression symptoms and other psychosocial variables [23], [24].

A second wave of assessment was conducted with surviving members of the LBC between 2008 and 2010. In this second wave, an attitudes to ageing measure was added to the assessment battery. The Attitudes to Ageing Questionnaire (AAQ) [12] was sent as a postal questionnaire to all available participants who had just completed wave two of the LBC1936 in April 2010. Physical and cognitive data were collected for some subjects immediately before AAQ completion, and for others up to three years previously.

### Participants: Sample Characteristics

There were 1,091 participants in wave 1 of the LBC 1936, and 866 participated in wave 2 [24]. Of these, 825 responded to the request to complete the AAQ (95.3% response rate): 802 returned a completed AAQ (92.6% completion rate; 44 of these were corrected by contacting the participants due to missing or multiple responses), an additional 10 were incomplete, one returned blank, three withdrew from the study, two subjects had died, and seven refused to complete the questionnaire.

Of the 802 participants, 414 (51.6%) were male, mean age 74.0 years (SD 0.28, range 73.4 to 74.7 years); all were Caucasian and English speaking, and community-dwelling with no severe acute physical or mental illness, or cognitive impairment. Mini mental state examination (MMSE) scores were on average (mean) 28.8 (SD 1.4, range 22 to 30). Social class distribution [25] was: social class I (Professional e.g. lawyer, doctor, clergyman, professional engineer) n = 152 (19.0%); II (Intermediate e.g. proprietor of business, trained nurse, artist) n = 296 (36.9%); III non-manual (Skilled e.g. clerk, policeman) n = 174 (21.7%); III manual (Skilled e.g. miner, chauffeur) n = 134 (16.7%); IV (Partly skilled e.g. fisherman, carter, stoker, conductor) n = 27 (3.4%); V (Unskilled e.g. labourer, railwayman, watchman) n = 5 (0.6%).

### The Attitudes to Ageing Questionnaire (AAQ)

The AAQ [12] was designed for completion by older people themselves and was developed following a robust psychometric procedure piloted with 1356 older people in 15 centres across the world and later field-tested with 5566 older people in 20 centres across the globe (including centres in Eastern and Western Europe, Asia (China and Japan) and North and South America). The mean age of the field trail sample was 72.53 (SD: 7.90). Almost 90% of the opportunistic sample lived in their own home or with family members. The 24 items of the AAQ scale are scored on a five-point Likert scale (1 =  strongly disagree, 5 =  strongly agree). Development of this AAQ followed a coherent, logical and empirical process taking full account of contemporary gerontological theory and both modern and classical psychometric analytical methods [12]. Exploratory factor analysis combined with an Item Response Theory approach using Rasch analysis was used in determining three distinct subscales for the AAQ: (1) Psychosocial Loss; (2) Physical Change; and (3) Psychological Growth. Each domain includes eight items. The three subscales of the AAQ report reasonably good PSI (Person Separation Index) scores of .807, .809 and .738 respectively. The PSI score is a summary estimate of the true variance relative to the sum of this variance and the error variance. It is used as a reliability index for Item Response Theory (IRT) equivalent to Cronbach's alpha.

The Psychosocial Loss subscale measures the perceived negative experiences of ageing and functions as a proxy for negative attitudes to ageing where old age is seen primarily as a negative experience involving psychological and social loss. Physical Change focuses on items primarily related to health and the experience of ageing itself, therefore a subjective individualised psychological perspective on health is assessed. Psychological Growth is explicitly positive and could be summarised as ‘Personal Wisdom' as it recognises a lifespan development perspective on ageing as viewed by the individual. Thus, the three domain structure of the AAQ reflects both positive and negative aspects of ageing.

Mokken analysis requires all scores to be directed in the same way, therefore the responses to factors (2) and (3) were recoded (1 to 5, 2 to 4 etc.) so that higher mean scores (calculated from the Likert scores on the items) reflected more negative attitudes. This does not suggest that this cohort has a negative attitude to ageing, but is required to allow Mokken analysis to be performed on the whole dataset.

### Mokken scaling

Within the range of methods for IRT, Mokken scaling [26] is proving to be an increasingly useful tool for investigating hierarchies of items in multivariate databases [19]. Mokken scaling, unlike other methods of IRT, is non-parametric and thereby offers rigorous - but less restrictive - opportunities for selecting hierarchies of items than, for example, Rasch scaling or other parametric methods. Mokken scaling works by seeking unidimensional sets of items on the basis of Loevinger's coefficient (H) which is based on the extent to which pairs of items, as scored by respondents, conform to Guttman criteria. In a Guttman scale—which is deterministic in nature—any pair of items should be scored relative to one another consistently; in other words, of two items item *i* and item *j*, if item *j* represents more of the latent trait then item *i* (i.e. it is more ‘difficult’ in psychometric terms) then item *i* should always be more readily endorsed than item *j*. Where item pairs are not endorsed in the expected direction (i.e. where an individual endorses item *j* more readily than item *i*) then that is a Guttman error. In this sense, ‘*difficulty*’ means the ease with which an item is endorsed or agreed with by respondents and is indicated by the mean score of the item: more ‘*difficult*’ items have lower mean scores. Loevinger's coefficient is calculated for item H (Hi); item pair H (Hij) and for the overall scale (Hs). By this means, and based on the mean scores on items by individuals, a set of items can be identified. A strong scale is evident when Hs>0.5, with moderate and weak scales present at Hs>0.4 and Hs>0.3, respectively [27]. To determine whether the AAQ conforms to a hierarchical scale, and how this relates to the original scales derived by factor analysis, we performed Mokken scaling to investigate the hierarchies within the AAQ.

Data were entered into an SPSS version 20.0 database and converted into a format suitable for Mokken scaling analysis (MSA) using the commercially available Mokken Scaling Analysis for Polytomous items (MSP) for Word version 5.0 [27]. The reliability of scales obtained by MSP is shown by a test-retest procedure similar to Cronbach's alpha, with reliable scales showing test-retest reliability rho>0.7 and p<0.05 (Bonferroni corrected for multiple testing) [27]. Furthermore, Mokken scaling can select items that conform to the model of monotone homogeneity (MMH) [26], whereby the items score increases consistently as the latent trait increases; items not conforming to the MMH can be removed.

Using a method recommended by Hempker *et al*. (1995) [28] and Meijer and Baneke (2001) [29], and applied by Nader *et al*. (2012) [30] to explore the data for multiple dimensions, the MSA was applied using MSP by searching for scales using incremental H values starting at a lowerbound Hs = 0.05 and raising this by 0.05 increments. This procedure was continued until an appropriate balance was found between the number of reliable scales (rho>0.7) and an absence of reliable but trivial scales with three items or fewer.

The Mokken Scaling Analysis for Polytomous items (MSP) for Word version 5.0 is very convenient for selecting scales by increasing the lowerbound value of Hs but this package does not contain software for analysing IIO, therefore, the data were then converted to a format suitable for analysis using the MSA in the public domain software ‘R’ [31] in which MSA software is available [32] to search for IIO in the Mokken scaled items. Unidimensional sets of items can be analysed for invariant item ordering (IIO), which is a crucial property of hierarchical scales [33]. IIO is the property whereby items that are ordered on the basis of the mean items scores of a group of respondents are also responded to in the same order by all of the respondents at all levels of the latent trait. Lack of IIO does not invalidate the use of a set of unidimensional items with groups of respondents on the basis of Hs≥0.3; this condition is necessary to establish a Mokken scale. However H≥0.3 is insufficient to establish IIO [34] and IIO is established on the basis of a parameter called Htrans (H^T^). H^T^, which measures how close item response functions are (IRFs) are, is analogous to H with the same minimum values representing weak, moderate and strong IIO, respectively. As an adjunct to inspecting IIO and calculating H^T^, we also plotted the IRFs of item pairs to inspect these for relative spacing, clustering and overlap. In R the standard errors for the AAQ items and item pairs were generated and used to calculate 95% confidence intervals (CIs). For items the lowerbound 95% CI should include the lowerbound value of H (0.30) and for the item pairs the lowerbound 95% CI should not include 0 [35].

### Factor analysis

Data were also analysed using exploratory structural equation modelling (SEM). The data were first analysed using principal components analysis (PCA) in SPSS version 20.0 and then using AMOS version 20.0 for SEM. We determined how many components to extract using a combination of: the criterion of eigenvalues >1; inspection of the scree slope plot of eigenalues; and Monte Carlo parallel analysis (http://www.softpedia.com/get/Others/Home-Education/Monte-Carlo-PCA-for-Parallel-Analysis.shtml; retrieved 18 November 2008)). Oblique (oblimin) rotation was used to determine a factor solution using loadings >0.40 to indicate loadings of items on putative factors. The final factor solution was then entered into AMOS as a series of SEMs representing the loadings of the factors obtained by PCA as a first order confirmatory factor analysis model [36] and modification index (MI) values were used to select pairs of residual variances to correlate until a range of fit indices (the goodness of fit index (GFI), the adjusted goodness of fit index (AGFI) and the comparative fit index (CFI) and the root mean square error of approximation (RMSEA) were acceptable at >0.90 and <0.06, respectively, indicating model fit.

To test the concurrent properties of the AAQ and to compare with previous work [13] we correlated newly identified dimensions of the AAQ with a range of variables including personality, psychological morbidity, social class, physical disability and also inspected the sensitivity to gender.

## Results

### Mokken scaling

The outcome of increasing the lowerbound Hs in 0.05 increments is shown in Table 1. From 0.05 to 0.15 all of the items formed a single scale after which two reliable scales were formed at Hs = 0.20 and four scales were formed at Hs = 0.25. At Hs = 0.30 five scales were formed; two of which were unreliable and two items were excluded by the analysis. At Hs = 0.35 four reliable scales were formed but one of these contained only two items and five items were excluded by the analysis. It should be noted that, while they were generated in a different order by the analysis, two of the scales were identical at Hs = 0.30 and Hs = 0.35. Since no new information was being obtained and an increasing number of items were being excluded, the final solution to the Mokken scaling was evident at the lowest acceptable Hs of 0.30 and this is shown in Table 2 (note that the original wording of the items is presented, with (-) used to indicate the reverse scoring for Mokken analyses). Three scales were identified and of these, two are weak Mokken scales (Scale 1 and Scale 3) and one is a moderate Mokken scale (Scale 2). All of the scales are reliable and statistically significant and Scale 2 shows weak IIO; the scales are described here with suggested names describing their content.

**Table 1:** Partitioning of items across Mokken scales with increasing lowerbound values of Hs (n = 802).

Items	Hs 0.05	Hs 0.10	Hs 0.15	Hs 0.20	Hs 0.25	Hs 0.30	Hs 0.35
1	Scale 1	Scale 1	Scale 1	Scale 2	Scale 3	Scale 4	DNS
2	Scale 1	Scale 1	Scale 1	Scale 2	Scale 3	Scale 4	DNS
3	Scale 1	Scale 1	Scale 1	Scale 1	Scale 1	Scale 3	Scale 4
4	Scale 1	Scale 1	Scale 1	Scale 2	Scale 2	Scale 2	Scale 3
5	Scale 1	Scale 1	Scale 1	Scale 1	Scale 1	Scale 1	Scale 1
6	Scale 1	Scale 1	Scale 1	Scale 1	Scale 1	Scale 1	Scale 1
7	Scale 1	Scale 1	Scale 1	Scale 1	Scale 1	DNS	Scale 2
8	Scale 1	Scale 1	Scale 1	Scale 1	Scale 1	Scale 1	Scale 1
9	Scale 1	Scale 1	Scale 1	Scale 1	DNS	Scale 3	Scale 4
10	Scale 1	Scale 1	Scale 1	Scale 2	Scale 2	DNS	DNS
11	Scale 1	Scale 1	Scale 1	Scale 1	Scale 1	Scale 1	DNS
12	Scale 1	Scale 1	Scale 1	Scale 1	Scale 1	Scale 3	Scale 4
13	Scale 1	Scale 1	Scale 1	DNS	Scale 4	Scale 5	DNS
14	Scale 1	Scale 1	Scale 1	Scale 1	Scale 1	Scale 1	Scale 1
15	Scale 1	Scale 1	Scale 1	Scale 1	Scale 1	Scale 1	Scale 1
16	Scale 1	Scale 1	Scale 1	Scale 1	Scale 4	Scale 5	DNS
17	Scale 1	Scale 1	Scale 1	Scale 1	Scale 1	Scale 3	Scale 4
18	Scale 1	Scale 1	Scale 1	Scale 2	Scale 2	Scale 2	Scale 3
19	Scale 1	Scale 1	Scale 1	Scale 2	Scale 2	Scale 2	Scale 3
20	Scale 1	Scale 1	Scale 1	Scale 1	Scale 1	Scale 3	Scale 4
21	Scale 1	Scale 1	Scale 1	Scale 2	Scale 2	Scale 2	Scale 3
22	Scale 1	Scale 1	Scale 1	Scale 1	Scale 1	Scale 3	Scale 4
23	Scale 1	Scale 1	Scale 1	Scale 1	Scale 1	Scale 1	Scale 1
24	Scale 1	Scale 1	Scale 1	Scale 1	Scale 1	Scale 1	Scale 2
(Reliability)	Scale 1 (0.87)	Scale 1 (0.87)	Scale 1 (0.87)	Scale 1 (0.85)	Scale 1 (0.84)	Scale 1 (0.80)	Scale 1 (0.79)
				Scale 2 (0.75)	Scale 2 (0.73)	Scale 2 (0.74)	Scale 2 (0.78)*
					Scale 3 (0.50)*	Scale 3 (0.78)	Scale 3 (0.74)
					Scale 4 (0.45)*	Scale 4 (0.45)*	Scale 4 (0.78)
						Scale 5 (0.45)*	

See Table 2 for item labels.

* =  too few items to form a scale.

DNS = did not scale.

**Table 2:** Mokken scaling of the Attitudes to Ageing Questionnaire with items ordered according to their mean score (n = 802).

Item	Label	Mean	Hi	AAQ Factor	Mokken Scale
7	(-) It is important to take exercise at any age	1.54	0.18	2 Physical Change	DNS
20	I don't feel involved in society now that I am older	1.77	0.43	1 Psychosocial Loss	3 Exclusion
17	As I get older I find it more difficult to make new friends	1.78	0.40	1 Psychosocial Loss	3 Exclusion
22	I feel excluded from things because of my age	1.85	0.41	1 Psychosocial Loss	3 Exclusion
9	I find it more difficult to talk about my feelings as I get older	1.86	0.35	1 Psychosocial Loss	3 Exclusion
15	I am losing my physical independence as I get older	1.89	0.34	1 Psychosocial Loss	1 Vitality
12	I see old age mainly as a time of loss	1.90	0.36	1 Psychosocial Loss	3 Exclusion
6	Old age is a depressing time of life	2.05	0.34	1 Psychosocial Loss	1 Vitality
5	(-) There are many pleasant things about growing older	2.09	0.32	3 Psychological Growth	1 Vitality
3	Old age is a time of loneliness	2.21	0.39	1 Psychosocial Loss	3 Exclusion
21	(-) I want to give a good example to younger people	2.2.5	0.48	3 Psychological Growth	2 Legacy
4	(-) Wisdom comes with age	2.30	0.39	3 Psychological Growth	2 Legacy
2	(-) It is a privilege to grow old	2.38	0.20	3 Psychological Growth	DNS
11	(-) I don't feel old	2.52	0.32	2 Physical Change	1 Vitality
24	(-) I keep myself as fit and active as possible by exercising	2.52	0.32	2 Physical Change	1 Vitality
13	(-) My identity is not defined by my age	2.55	0.18	2 Physical Change	DNS
10	(-) I am more accepting of myself as I have grown older	2.57	0.22	3 Psychological Growth	DNS
1	(-) As people get older they are better able to cope with life	2.59	0.19	3 Psychological Growth	DNS
8	(-) Growing older has been easier than I thought	2.62	0.41	2 Physical Change	1 Vitality
23	(-) My health is better that I expected for my age	2.64	0.42	2 Physical Change	1 Vitality
18	(-) It is important to pass on the benefits of my experience to younger people	2.72	0.52	3 Psychological Growth	2 Legacy
16	(-) Problems with my physical health do not hold me back from doing what I want to do	2.79	0.21	2 Physical Change	DNS
19	(-) I believe my life has made a difference	2.86	0.42	3 Psychological Growth	2 Legacy
14	(-) I have more energy now than I expected for my age	2.89	0.46	2 Physical Change	1 Vitality

(-) used to indicate that analyses reverse-scored these items.

DNS = did not scale.

AAQ Factor  =  Factor as derived in original factor analyses [12].

For mean scores, scores are on Likert scale, 1 =  strongly disagree, 3 =  neither agree nor disagree, 5 =  strongly agree; a high score indicates a more negative attitude towards ageing.

***Mokken Scale 1: Vitality/Person focussed ageing***: Hs = 0.37; Rho = 0.80; p = 0.00012; H^T^ = 0.23.

***Mokken Scale 2: Legacy/Social value***: Hs = 0.46; Rho = 0.74; p = 0.00031; H^T^ = 0.31;

***Mokken Scale 3: Exclusion/Social role***: Hs = 0.39; Rho = 0.78; p = 0.00047; H^T^ = 0.07.

#### Scale 1

‘Vitality’ – this scale contains eight items (Table 2) related to physical and psychological aspects of ‘person-focussed ageing’. Taking the reverse scoring of items into account, the scale describes a hierarchy of loss of a positive attitude to the physical and psychological aspects of ageing which could be summarised in the concept ‘ageing has been better than I expected.’ The most readily endorsed concept is that of having more energy than a person expected (‘I have more energy now than I expected for my age’ - reverse scored - from the Physical Change dimension) which is a very general aspect of ageing, through further physical aspects such as health and physical fitness with psychological aspects interspersed (from the Physical Change and Psychosocial Loss dimensions): ‘I don't feel old’; ‘Old age is a depressing time of life’. The least readily endorsed concepts express pleasantness (‘There are many pleasant things about growing older’ and the expression that it is ‘depressing’) with the least readily endorsed item being ‘I am losing my physical independence as I get older’. Therefore, the hierarchy of endorsement is from general items about ageing and the unexpected preservation of vitality, through acknowledging some negative aspects of ageing, to the loss of independence. People most easily report physical loss, but less easily endorse statements about psychosocial loss. This scale is only a weak scale and does not show IIO which means that it is useful in ordering respondents but that they do not necessarily all respond to the items in the scale in the same order. Inspection of the item pair plots of the IRFs for this scale showed that, while these were not overlapping, there was a cluster of items (items 8, 11, 23 & 24) in the scale which possibly accounted for the value of H^T^ which was too low to assume IIO. The 95% CI for item 15 includes 0 for its pairing with other items; however, these items are not included in Scale 1.

#### Scale 2

‘Legacy’ – this scale contains four items related to the impact of a person's ageing on other people, their ‘social value’. It describes a hierarchy of concern related to being noticed and having wisdom and example to pass on, e.g. in the question ‘how much has ageing made me count?’ The most readily endorsed concept is about having made a difference (‘I believe my life has made a difference’) through importance in passing thing on, being wiser, to the least readily endorsed item ‘I want to give a good example to younger people’. Therefore, the hierarchy of endorsement is from feeling a sense of making a difference to feeling that you want to pass things on. This scale shows weak IIO (H^T^>0.3) which indicates that all respondents at any level of attitude may respond to these items in the same way. However, inspection of the item pair plots Scale 2 shows that the IRF for item 19 is far from the remaining items and this could be exaggerating the measurement of IIO as shown recently [37], [38]. Items 18, 19 and 21 in this scale had 95% CIs which included 0 with items which were not included in Scale 2.

#### Scale 3

‘Exclusion’– this scale contains six items related to ‘social role’ particularly social exclusion. It describes a hierarchy of exclusion related to loneliness, loss and lack of involvement which could be summarised in the question ‘how much has ageing excluded me?’ The most readily endorsed concept is about old age being lonely (‘Old age is a time of loneliness’) through further concern about loss, exclusion and lack of friends with the least readily endorsed concept being about involvement (‘I don't feel involved in society now that I am older’). Of note, by endorsement we do not mean to imply agreement with these statements, simply the *extent* to which they were agreed with; for example, most people agreed with the statement ‘Old age is a time of loneliness’, therefore this is the most endorsed concept within that scale. Therefore, the hierarchy of endorsement is from a general concern that old age is a time of loss and loneliness through to specific expressions of exclusion, lack of friends and lack of involvement. Inspection of item pair plots showed that all of the IRFs, while not overlapping, were clustered for this scale which explains the very low value of H^T^ and the apparent lack of IIO. Item 17 had a 95% CI which included 0 with an item not included in Scale 3.

The following six items did not scale because they did not achieve the lowerbound value of 0.30 for Hi meaning that, with other items, they caused too many Guttman errors and their retention in the scale would lower the values of Hij and the overall Hs. These items were: (from the Physical Change dimension) ‘It is important to take exercise at any age’; ‘My identity is not defined by my age’; ‘Problems with my physical health do not hold me back from doing what I want’; and (from the Psychological Growth dimension) ‘As people get older they are better able to cope with life’; ‘It is a privilege to grow old’; ‘I am more accepting of myself as I have grown older’. Several items pairs had lowerbound 95% CIs which included 0, these were: items 9 and 18; items 9 and 21; items 15 and 19; items 15 and 21; and items 16 and 17. Some of these items have been considered individually above.

In summary, the Mokken scaling of the AAQ shows that there is a hierarchy of responses within three separate scales, referring to vitality/person-focussed ageing (hierarchy from physical to psychosocial), legacy/social value (from feeling a sense of making a difference to feeling that you want to pass things on) and exclusion/social role (hierarchy from general to specific instances). These differ from the dimensions determined by factor analysis.

### Factor analysis

**Figure 1:**
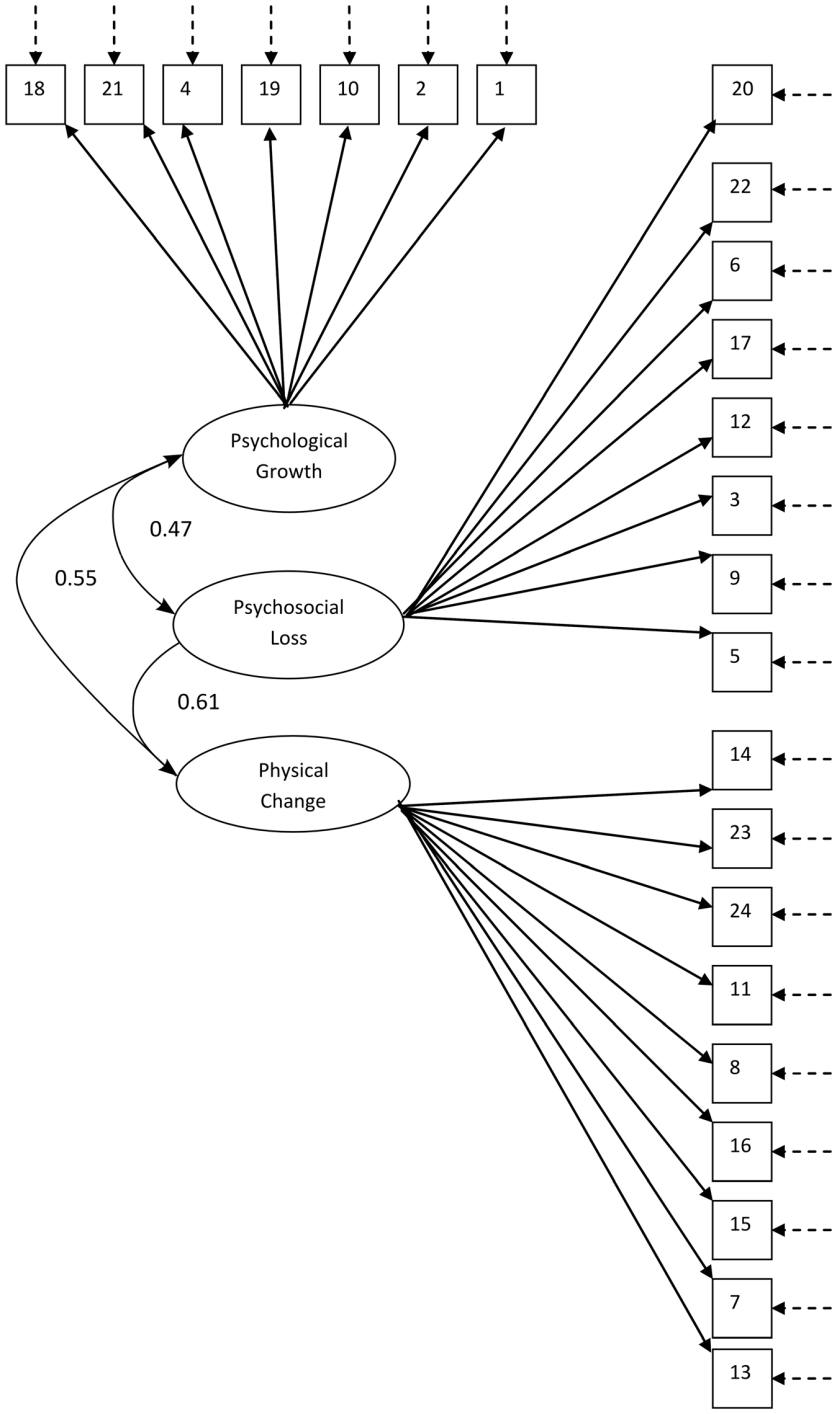
Factor structure of the AAQ scale. Diagrammatic representation of structural equations representing hypothesised model of the relationship between variables in the AAQ. Squares represent the AAQ variables, ovals represent first-order latent variables. Standardised regression weights of first-order factors on second order stress factor are shown; standardised regression weights of SINS items on first-order factors are shown in Table 5; broken arrows represent error variance; intercorrelated error variances are shown in Table 5.

Inspection of eigenvalues suggested a five-factor solution explaining 51% of the post-rotational variance, but the parallel analysis suggested a maximum of four factors explaining 46.5% of the post-rotational variance, and the scree slope method between three to four factors, the three factor solution explaining 41% of the post-rotational variance. Both a four- and a three-factor solution were inspected following oblique rotation; the four-factor solution produced one trivial factor with only two items loading and the distribution of items across factors was hard to interpret. However, the three factor solution (Table 3) produced a reasonably simple solution (one whereby loadings were high on putative factors with low loadings elsewhere). Some cross-loading was evident—often a reason to remove items and re-rotate—but the distribution of items, with two exceptions, showed remarkable congruence with the factor solution reported by Laidlaw et al, 2007. The two exceptions were the items ‘My identity is not defined by my age’, previously loading on the Physical Change factor and now loading on the Psychosocial Loss factor, and ‘I am losing my physical independence as I get older’, previously loading on the Psychosocial Loss factor and now loading on the Physical Change factor. The first order factor structure is shown in Figure 1 and the correlated residuals are shown in Table 4. The proposed solution and fit indices are shown in Tables 5 and 6, respectively. The GFI, AGFI and CFI all exceeded 0.90 and the RMSEA was lower than 0.06. With the reduction in degrees of freedom in the model, the value of Chi-square reduced, but the high, significant value of Chi-square—which should ideally be low and non-significant—in the final model is probably due to the large sample size.

**Table 3:** Principal components analysis with oblimin rotation of the AAQ.

Item*	Commonality	First principal unrotated component	Factor loading 1^a^	Factor loading 2^a^	Factor loading 3^a^
18	0.552	0.429	**738**	121	188
21	0.510	0.439	**713**	152	198
4	0.423	0.405	**647**	171	160
19	0.426	0.471	**646**	256	212
10	0.324	0.451	**552**	187	314
2	0.255	0.430	**485**	236	272
1	0.234	0.398	**465**	183	277
20	0.525	0.513	156	**722**	208
22	0.463	0.553	203	**678**	300
6	0.494	0.648	346	**670**	394
17	0.459	0.498	229	**669**	173
12	0.446	0.480	110	**664**	236
3	0.435	0.563	271	**650**	295
9	0.399	0.419	130	**624**	134
5	0.321	0.562	411	**443**	396
14	0.656	0.637	290	288	**809**
23	0.510	0.581	303	259	**711**
24	0.409	0.511	182	284	**635**
11	0.386	0.499	324	180	**605**
8	0.434	0.647	451	418	**568**
16	0.322	0.435	226	164	**564**
15	0.443	0.511	030	490	**543**
7	0.252	0.328	078	131	**492**
13	0.199	0.380	229	171	**438**
**Cronbach's alpha**		0.86	0.74	0.80	**0.78**

*See Table 2 for the labelling of items.

a For clarity loadings on putative factors are shown in bold and only the places after the decimal point are shown.

**Table 4:** Correlation between error variances.

Error	Pair*	Correlation
24	7	0.469
20	17	0.299
6	8	0.275
18	21	0.286
5	2	0.204
12	15	0.210
14	23	0.290
2	1	0.165
18	10	0.169

*See Table 2 for the labelling of items.

**Table 5:** Standardised regression weights of SINS-CN items on first-order factors and squared multiple correlations of error variances.

Item	Psychological Growth	Psychosocial Loss	Physical Change	Unique Variance
18	0.642			0.412
21	0.578			0.334
4	0.559			0.313
19	0.584			0.341
10	0.525			0.276
2	0.371			0.138
1	0.396			0.157
20		0.565		0.319
22		0.600		0.360
6		0.697		0.486
17		0.526		0.277
12		0.560		0.314
3		0.647		0.418
9		0.491		0.241
5		0.473		0.224
14			0.739	0.547
23			0.636	0.404
24			0.507	0.257
11			0.536	0.287
8			0.623	0.388
16			0.477	0.227
15			0.523	0.274
7			0.298	0.089
13			0.385	0.148

**Table 6:** Fit indices for confirmatory factor analysis of the SINS-CN scale (values prior to restriction imposed on the model are shown in brackets).

Fit index	Value
GFI	0.928 (0.882)
AGFI	0.910 (0.858)
CFI	0.903 (0.809)
RMSEA	0.041 (0.050)

Chi-Square 729.980; df = 240 (1213.291; df = 249); p<0.0001.

GFI = goodness of fit index; AGFI = adjusted goodness of fit index; CFI = comparative fit index; RMSEA = root mean square error of approximation.

Pearson's correlation between the newly identified dimensions of the AAQ and a range of variables is shown in Table 7. Broadly speaking, the concurrent relationships observed by Shenkin *et al*, 2014 hold in our study. For example, higher levels of disability are negatively correlated with Vitality and positively correlated with Exclusion; likewise higher levels of anxiety and depression; neuroticism is negatively correlated with Vitality and positively correlated with Exclusion and levels of extraversion, openness, agreeableness and conscientiousness correlate in the expected directions. Females have a more negative experience of Vitality (p = 0.012).

**Table 7:** Pearson's correlation between AAQ dimensions and a range of variables.

	Vitality	Legacy	Exclusion
Social class	0.074*	0.188**	0.037
Townsend's disability scale	0.285**	0.005	0.148**
HADS anxiety	−0.182**	0.019	0.232**
HADS depression	−0.427**	0.166**	0.432**
NEO-FFI neuroticism	−0.292**	0.103**	0.349**
NEO-FFI extraversion	0.311**	0.293**	−0.328**
NEO-FFI openness	0.137**	−0.016	−0.131**
NEO-FFI agreeableness	0.202**	0.190**	−0.263**
NEO-FFI conscientiousness	0.298**	0.329**	−0.269**

HADS = Hospital Anxiety and Depression scale; NEO-FFI = NEO Five Factor Index;

* = p<0.05; ** = p<0.01;

Note: in this table, higher scores on the AAQ dimension of Vitality indicates a more positive attitude; higher scores on Legacy indicate a more positive attitude; and higher scores on Exclusion indicate a more negative attitudes towards ageing.

## Discussion

The aim of this study was to investigate the AAQ, using Mokken scaling analysis, for the existence of hierarchical scales within its dimensions. Understanding of the hierarchies reported by healthy community dwelling older people's attitudes to ageing helps to increase understanding of the items which are seen as most important by older people themselves. Recent work using Mokken scaling, summarised by Watson *et al*. 2012, shows that hierarchical scales exist within several well-established scales used to measure, for example, psychological morbidity but also activities of daily living and quality of life. These scales often cut across existing structures demonstrated using factor analysis, as exemplified by the Mokken scaling of the 30 item General Health Questionnaire where the resulting 9-item scale contains items from several of the dimensions of the original scale [20]. The Mokken scales of the present study have face validity in the sense that the hierarchical arrangement of items is interpretable in terms of the latent trait being measured and the utility of the scale is increased because the score on a Mokken scale is a measure for the order of the latent trait: scores are related to specific sets of items. Taking the General Health Questionnaire (GHQ) as an example [20], nine items from the GHQ-30 form a Mokken scale running, in terms of endorsement, from items indicating general psychological distress (‘Been (un)able to face up to your problems?’) to items indicating suicidal ideation (‘Felt that life isn't worth living?’). The same phenomenon is observed in the Clinical Outcomes in Routine Evaluation-Outcome Measure [39].

The requirement of Mokken scaling to have all items in the AAQ scored in the same direction may cause some confusion with interpretation of the results. By reverse scoring items we do not in any way mean to imply a negative attitude to ageing, this is merely a requirement of the statistical method. For example, many respondents disagreed with the questions with a negative perspective on attitudes to ageing, (e.g. strongly disagreeing with “I am losing my physical independence as I get older” [mean score 1.89] and with “I have more energy now than I expected for my age” [reverse scored, mean score 2.89]). However, in the context of Mokken analysis, the strength of the endorsement (i.e. the higher the mean value) is the most important factor in establishing the hierarchy.

The original AAQ revealed three dimensions using factor analysis: Psychosocial Loss; Physical Change; and Psychological Growth, with mostly positive attitudes to ageing. Analysis of the AAQ in individual national populations [14]–[16] confirmed this structure, but Kalfoss *et al*., 2010 and Chachamovich *et al*, 2008 noted minor differences in the distributions of responses in the Canadian and Norwegian groups. In our study exploratory SEM supported the original factor structure of the AAQ reported by Laidlaw *et al*., 2007. With the exception of two items, the structure was identical and, in fact, the items which loaded on different factors were more congruent with the factors in the present study: ‘My identity is not defined by my age’, now loading on the Psychosocial Loss factor, and ‘I am losing my physical independence as I get older’ now loading on the Physical Change factor.

In the present analysis, Mokken scaling uses 18 items also revealing three dimensions, two of which are restricted to items from the existing dimensions of Psychological Growth and Psychosocial Loss and a third which is composed mainly of items from the Physical Change dimension but including items from the other two dimensions. The dimensions have been described in detail in the Results section and the purpose here is to describe the added value of extracting a sub-set of 18 items, from the original 24, into three dimensions, one of which combines items from the dimensions established by factor analysis. Scale 1, labelled ‘Vitality/Person-focussed ageing’, appears to describe aspects across all of the existing dimensions related to frailty. According to the order of items in Scale 1, attitudes to vitality begin with physical factors (e.g. health) and ends with psychosocial aspects such as depression. Scale 2, labelled ‘Legacy/Social value’, encompasses some aspects of Psychological Growth and appears to describe aspects of legacy whereby an positive attitude to ageing is accompanied by feeling that your life was worth living and you have value to pass to the next generation. Finally, Scale 3, labelled ‘Exclusion/Social role’, encompasses Psychosocial Loss related to exclusion which begins with feelings of loneliness but ends with feeling that you are no longer involved in what is going on. Therefore, our analysis shows that there are both similarities and differences between the solutions obtained for multivariate analysis of the AAQ using factor analysis and Mokken scaling. The similarities point to the strength of the AAQ in terms of its conceptual development and its ability to measure Psychological Growth and Psychosocial Loss and this also points to the strength of the factor analytic approach in identifying unidimensional sets of items in the original item pool. The ‘added value’ of the Mokken scaling analysis here is twofold: first, within the Psychological Growth and Psychosocial Loss factors of the AAQ it has identified, more specifically, sets of items focusing on narrower aspects of these two major dimensions—Legacy and Exclusion; second, Mokken scaling has identified a hitherto unidentified dimension of Vitality that is composed of items from all three of the original dimensions of the AAQ. Analysis of correlation with range of variables studied by Shenkin et al, 2014, and difference between males and females show that the dimensions of the AAQ identified using Mokken scaling show some construct validity. This suggests that these new scales, which probably need some development, show promise in the study of attitudes towards ageing among older people.

All three of the Mokken scales were identified on the basis of their ability to order respondents, and claims for IIO are, at best, weak. Some manipulation of the present scales such as the removal of specific items from the Vitality and Exclusion scales may improve IIO. It is likely that the Legacy scale already shows artificially high IIO and may require further development. Further work would be required either to refine items or replace them in the Mokken scales if their exclusion leads to construct underrepresentation. This could lead to an alternative, possibly shorter, version of the AAQ with a hierarchy of questions to rapidly identify people with specific attitudes to ageing. This is important because participants with a more positive attitude to ageing engaged in more healthy behaviours and report higher rates of wellbeing [6]. The direction of causality is unknown, and it is therefore possible that attitudes to ageing could be targeted for interventions which could, in turn, perhaps improve older people's health [4].

## Conclusions

In this cohort of relatively healthy, community dwelling older people, Mokken scaling has revealed new dimensions within the AAQ and, as a result of the hierarchical nature of the technique, relates levels on these dimensions to specific items. In part, this analysis supports the original factor analysis of the AAQ which did not address the hierarchy of items. Mokken scaling of the AAQ demonstrated that within three aspects of attitudes to ageing, responses were in a hierarchical fashion: relating to vitality there was a hierarchy from physical to psychosocial; relating to legacy there was a hierarchy from individual contributions to passing things on, and relating to exclusion, there was a hierarchy from general to specific instances. This helps us to understand aspects of ageing that are important to older people, and may provide a novel target for intervention to improve health and well-being.
