# Correction: The Attitudes to Ageing Questionnaire: Mokken Scaling Analysis

**DOI:** 10.1371/journal.pone.0108766

**Published:** 2014-09-18

**Authors:** The PLOS ONE Staff

The legends for Table 5, Table 6, and Figure 1 incorrectly refer to “SINS” rather than “AAQ” items. The authors have provided corrected legends below.

**Figure 1:**
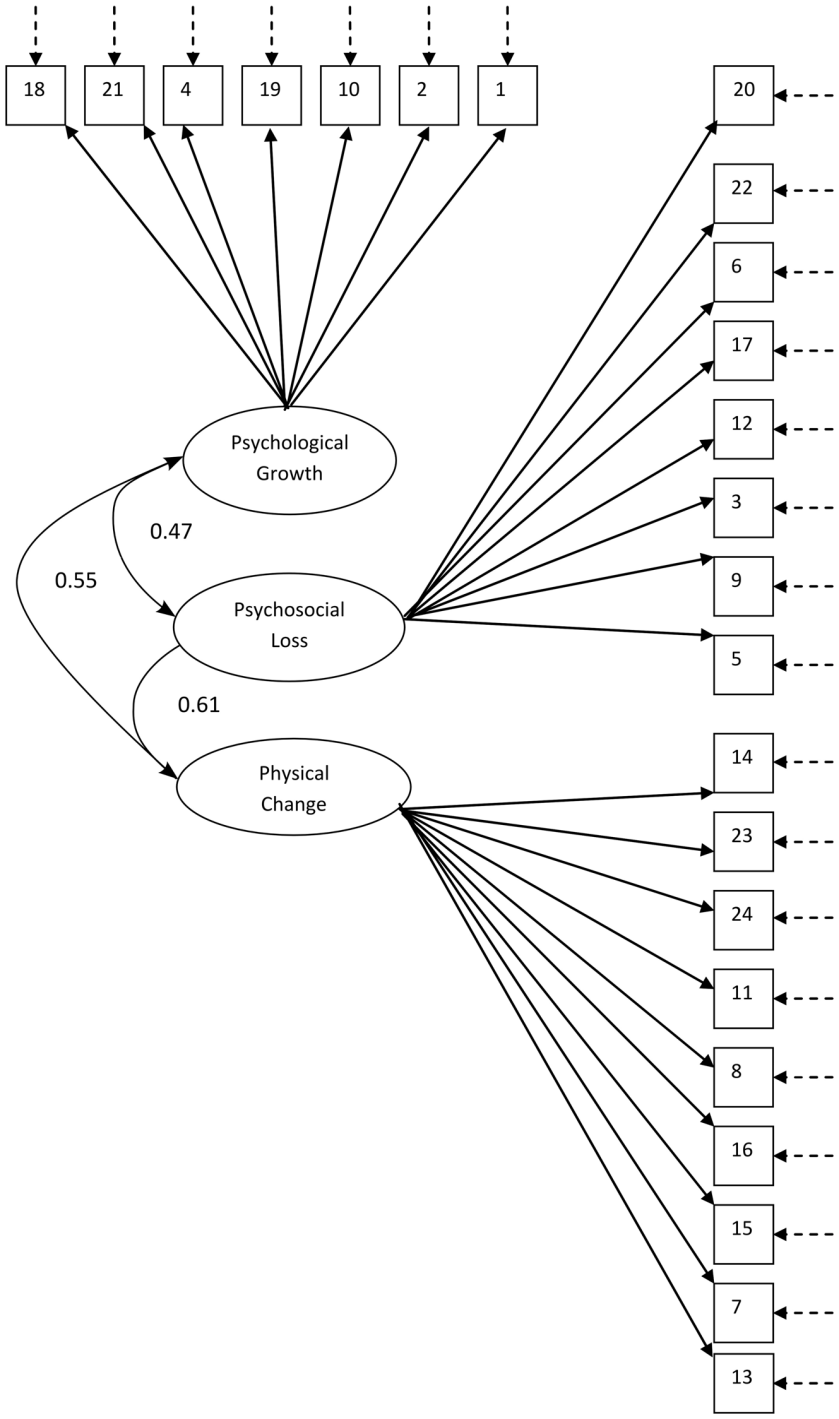
Factor structure of the AAQ scale. Diagrammatic representation of structural equations representing hypothesised model of the relationship between variables in the AAQ. Squares represent the AAQ variables, ovals represent first-order latent variables. Standardised regression weights of first-order factors on second order stress factor are shown; standardised regression weights of AAQ items on first-order factors are shown in Table 5; broken arrows represent error variance; intercorrelated error variances are shown in Table 5.

**Table 5:** Standardised regression weights of AAQ items on first-order factors and squared multiple correlations of error variances.

Item	Psychological Growth	Psychosocial Loss	Physical Change	Unique Variance
18	0.642			0.412
21	0.578			0.334
4	0.559			0.313
19	0.584			0.341
10	0.525			0.276
2	0.371			0.138
1	0.396			0.157
20		0.565		0.319
22		0.600		0.360
6		0.697		0.486
17		0.526		0.277
12		0.560		0.314
3		0.647		0.418
9		0.491		0.241
5		0.473		0.224
14			0.739	0.547
23			0.636	0.404
24			0.507	0.257
11			0.536	0.287
8			0.623	0.388
16			0.477	0.227
15			0.523	0.274
7			0.298	0.089
13			0.385	0.148

**Table 6:** Fit indices for confirmatory factor analysis of the AAQ scale (values prior to restriction imposed on the model are shown in brackets).

Fit index	Value
GFI	0.928 (0.882)
AGFI	0.910 (0.858)
CFI	0.903 (0.809)
RMSEA	0.041 (0.050)

Chi-Square 729.980; df = 240 (1213.291; df = 249); p<0.0001.

GFI = goodness of fit index; AGFI = adjusted goodness of fit index; CFI = comparative fit index; RMSEA = root mean square error of approximation.

## References

[1] Shenkin SD, Watson R, Laidlaw K, Starr JM, Deary IJ (2014) The Attitudes to Ageing Questionnaire: Mokken Scaling Analysis. PLoS ONE 9(6): e99100 doi:10.1371/journal.pone.0099100 24892302 10.1371/journal.pone.0099100 PMC4043998

